# Tissue specific microenvironments: a key tool for tissue engineering and regenerative medicine

**DOI:** 10.1186/s13036-017-0077-0

**Published:** 2017-11-16

**Authors:** Patrick C. Sachs, Peter A. Mollica, Robert D. Bruno

**Affiliations:** Medical Diagnostic and Translational Sciences, College of Health Science, Old Dominion University, Norfolk, VA 23529 USA

## Abstract

The accumulated evidence points to the microenvironment as the primary mediator of cellular fate determination. Comprised of parenchymal cells, stromal cells, structural extracellular matrix proteins, and signaling molecules, the microenvironment is a complex and synergistic edifice that varies tissue to tissue. Furthermore, it has become increasingly clear that the microenvironment plays crucial roles in the establishment and progression of diseases such as cardiovascular disease, neurodegeneration, cancer, and ageing. Here we review the historical perspectives on the microenvironment, and how it has directed current explorations in tissue engineering. By thoroughly understanding the role of the microenvironment, we can begin to correctly manipulate it to prevent and cure diseases through regenerative medicine techniques.

## Background

“We are drowning in information but starved for knowledge.” – John Naisbitt. *Megatrends*.

Perhaps the most fundamental question in all of biology is how a genetic clone can produce the vast array of cellular populations needed to sustain life in multicellular organisms. The elucidation of epigenetic mechanisms that regulate gene expression provides a molecular framework for understanding cell fate determination. However, questions persist as to how cells “know” to adopt specific epigenetic profiles during development. While these are questions of developmental biology, the answers are of vital importance for regenerative medicine and tissue engineering as well.

We now know cells respond to signals within their environment to induce differentiation down specific lineages. Isolation and characterization of embryonic stem cells allowed for the precise identification of discrete factors sufficient to induce differentiation down major cellular lineages of the body [1]. Fundamental to this discussion, however, is the now accepted principal that cellular differentiation is not a one way street, and, by extension, cell fate is not a terminal state. This is most notably demonstrated by the Nobel Prize winning works of John Gurdon and Shinya Yamanaka whose combined experiments (performed decades apart) demonstrated that cells could be “reprogrammed” to become pluripotent [2–4]. These findings, combined with our understanding of the power of extracellular signals, and epigenetic profiles to induce differentiation, will provide researchers with essential tools to probe the processes of tissue and organ development.

Still, as is often the case in biology, the more we know, the less we understand. Moreover, in the fast moving technology driven age we are in, important pieces of data often get overlooked or forgotten. While an extensive review of all of the relevant *information* concerning fate determination is not feasible, this review will seek to highlight historical data that informs our *knowledge* of cell fate determination. Specifically, we will review the evidence demonstrating the microenvironmental control of cell fate and describe how these advances are, or could be, exploited for tissue engineering and regenerative medicine.

## Part I: On the fate of cells

“The development of an organism … may be considered as the execution of a 'developmental program' present in the fertilized egg. … A central task of developmental biology is to discover the underlying algorithm from the course of development.” — Aristid Lindenmayer, *Automata, Languages, Development* (1976)

### Cellular differentiation and plasticity:

Developmental biologists have long grappled with two alternative, although not incongruous perspectives of cellular differentiation: cellular (intrinsic) and microenvironmental (extrinsic). Experimental evidence supports a role for both. From a cellular perspective, it is clear that epigenetic alterations lead to discrete gene expression profiles, and in turn, distinct cellular functions of cells. However, the role of the microenvironment in controlling these epigenetic profiles is also well established. A cell can obviously not accomplish anything that requires tools not encoded within its genome, but the cell seems dependent on its environment for feedback on how to proceed. Modern biology has provided a wealth of information regarding the individual pieces of this developmental puzzle. The challenge going forward is to develop the knowledge necessary to put the puzzle together, for the interplay between genetics, epigenetics, and the microenvironment is the “underlying algorithm” [5] of development.

The famous metaphor for cellular differentiation is a ball rolling down a bumpy landscape as described by Conrad Waddington in 1957 [6]. In this model, cells interact with an epigenetic landscape that guides them down specific differentiation paths (creodes) to terminal differentiation. Waddington’s model allows for variability in the differentiation process, but it does imply that differentiation is overall unidirectional—i.e. the ball never rolls back up hill. However, a mere five years after Waddington published his work, John Gurdon demonstrated that transplantation of the nucleus of a mature intestinal frog cell into a enucleated egg could produce a normal tadpole. Sixty-four years later, Shinya Yamanaka demonstrated that ectopic expression of 4 genes (Sox 2, Oct-4, Klf4, and c-Myc) could convert adult differentiated cells into induced pluripotent stem cells (iPSCs) [7]. Importantly, only a transient exogenous expression of these genes was required to drive the cells back to a pluripotent state, at which time the iPSC cells were capable of generating expression profiles sufficient to maintain their pluripotency. This provides the molecular tools necessary to drive Waddington’s model in reverse, sending the ball back to the top of the hill to differentiate once again. In fairness to Waddington, he was discussing what “is” in development, not what “could be.” However, from the perspective of tissue engineering and regenerative medicine, “what could be?” is the key question.

### Mesenchymal control of form and function:

Beyond nuclear transfer and ectopic gene expression, cumulative evidence demonstrates that the cellular microenvironment can mediate cell fate determination [8–10]. Classic experiments demonstrated the role of inductive mesenchyme from various tissues types in controlling both the form and function of developing tissues. An exceptional example of this is the ability of molar mesenchyme of mice to induce tooth development in chick epithelium as described by Kollar and Fisher in 1980 [11]. In these experiments, epithelial cells from the pharyngeal arches of E5 chic embryos were combined with mesenchymal cells from mandibular molars of E16–18 CD-1 mice and grafted into the eyes of athymic nude mice. The results indicated that the chick epithelium differentiated to form normal tooth structures that deposited enamel matrix. Nearly 30 years later, the use of mesenchymal cells to direct tooth formation for regenerative medicine purposes was described by Tsuji and colleagues [12]. Their strategy was remarkably similar to that employed by Kollar and Fisher. Disappointingly, however, they failed to cite Kollar and Fisher’s work in any of their related papers. This suggests that the authors had to rediscover this process on their own. Perhaps this helps explains the almost 3 decade gap in developing a translational application for this finding. Similar results to those described for molar mesenchyme were found in experiments demonstrating control of cell fate by mesenchyme to drive feather/scale and prostatic cellular differentiation [13, 14]. These results demonstrate the extensive power of the stromal microenvironment. If chic epithelium could be coerced into forming teeth, then perhaps the limits of microenvironmental induced differentiation are only limited by the genetic information available in the target cells.

In an earlier experiment, Sakakura and colleagues found that E14 salivary mesenchyme would direct E16 mammary epithelial cells to grow with a characteristic salivary morphology within the kidney capsule of syngeneic hosts [15]. Despite the salivary gland morphology, the resulting structures retained mammary epithelial cytodifferentiation, evidenced by their milk protein production during pregnancy [15]. Therefore, while oral mesenchyme was sufficient to direct complete cytodifferentiation of epithelial cells to teeth, embryonic salivary mesenchyme was sufficient to direct morphology but not cytodifferentiation of embryonic mammary epithelial cells. The contrast is an important highlight of the complexity of microenvironmental control of cell fate. Both the source of parenchymal cells, and the stromal/mesenchymal signals they are exposed to matter. The extent of the response is likely mediated by the epigenetic landscape present within the parenchymal cells prior to the interaction. In other words, specific epigenetic profiles may make a cell source incapable of responding to the microenvironment, while others may facilitate it. A simple analogy is that of a radio receiver and broadcast radio waves. The receiver can only process signals that are transmitted at frequencies to which it can be tuned. Similarly, the level of cellular response to a microenvironment is likely limited by both the signals presented and the cells ability to interpret and respond to those signals.

### The stem cell niche

The stem cell niche was conceptualized by Ray Schofield to explain the equal propensity of young and old bone marrow to graft in donor hosts [16]. The idea was that stem cells resided in protective tissue locales (niches). These niches protected the stem cells from differentiation, and rendered them effectively “immortal”, thus allowing them to continue to function when isolated from aged animals. Since that time, a great deal of experimental evidence has emerged to support the physical existent of stem cell niches in diverse experimental models [17–20]. The nature and function of the stem cell niche has been reviewed in detail before [8, 20], and are beyond the scope of this discussion. Rather, we will focus on the role the niche plays in fate determination and how this can be exploited in tissue engineering and regenerative medicine.

There is no strict definition of what actually constitutes a stem cell niche. In fact, the term “niche” is not even restricted to stem cells, as niches are associated with the progenitor cell function and maintenance as well [9, 18]. While the argument is largely semantic, some clarification is in order. The major functions of the niche are to prevent differentiation and coordinate asymmetric divisions to allow for self-renewal of the stem/progenitor cell. Essentially, anything that contributes to the maintenance and function of stem/progenitor cells could be identified as a component of the niche. This would include the broader microenvironment as it helps drive differentiation of stem/progenitor daughter cells, and therefore is vital for stem/progenitor cell function. In other words, the stem cell niche can be defined simply as the microenvironment in which the stem cell resides.

In mammalian tissues, the niche is likely a complex mixture of cellular interactions and signaling mediated through the extracellular matrix. However, a niche does not need to necessarily be complex. This may be especially true in developing tissues, where the niche is changing. For example, during development of the drosophilia midgut, evidence suggests progenitor cells expand symmetrically, and are maintained by a transient niche formed from a peripheral cell [19]. As the gland develops, the peripheral cell is lost, one progenitor is recruited to a permanent stem cell niche, and the others differentiate into enteroblasts. Another example occurs during T-lymphocyte division during the initiation of the adaptive immune response [17, 21]. In this case, the antigen presenting cell serves as a temporary niche to establish a division plane with the distal daughter cell becoming the memory T-cell and the proximal daughter cell undergoing amplification and terminal differentiation to produce effector T cells. In a more artificial system, Habbib et al. [22] demonstrated that a single localized signaling molecule, WNT3A, could drive asymmetric divisions and stem cell self-renewal of naïve pluripotent embryonic stem cells (ESCs). The ESCs were cultured in neuronal differentiation medium N2B27 on culture plates containing randomly distributed WNT3A tethered microbeads. The ESCs that were in contact with the WNT3A tethered beads divided asymmetrically with the proximal cell retaining pluripotency markers and the distal cell differentiating to an epiblast state. Those not in contact with a WNT3A bead underwent symmetric divisions with both daughter cells differentiating. Therefore, the localized WNT3A signal combined with differentiation inductive medium supplied a functional niche.

Problems of tissue engineering and regenerative medicine are rooted in the same problems of developmental biology (i.e. tissue/organ development). Therefore, understanding how a stem/progenitor cell niche is organized for tissue regeneration is important. However, the examples above serve to underscore that discrete signals can serve to coordinate early events in tissue development. This holds promise for engineering applications; however, determining how to harness the power of the niche is the key.

### Lessons on the stem cell niche from chimeric mammary glands

The stem cell niche brings us back to the dual perspectives of developmental biology: intrinsic vs. extrinsic. In other words, are the properties ascribed to tissue-specific stem /progenitor cells intrinsic to the cells themselves or to the niche in which they reside? Over the past decade, Dr. Gilbert Smith and colleagues have performed a series of interesting experiments using the mouse mammary gland model that probe this question [9, 18, 23–33]. The mammary gland of mice is regenerative. Any portion of the epithelial tree can recapitulate a new functional tree upon transplantation into mammary fat-pads of recipient animals that have had their endogenous epithelium surgically removed [8]. This can be achieved by transplanting either dispersed epithelial cells or tissue fragments. The regenerative process is mediated by stem and progenitor cellular functions [8] and is unaffected by age or reproductive history of the donor. Therefore, if the stem cell niche theory is correct, when dispersed mammary epithelial cells were transplanted, they must reform a functional niche to facilitate gland regeneration.

This allows for an interesting opportunity to test the capacity of the niche to control cell fate. Smith and colleagues combined non-mammary stem/progenitor cells with normal mammary epithelial cells and transplanted them into the epithelium divested fat pads of recipient mice. The experimental conditions tested whether non-mammary stem cells could be incorporated into mammary niches, and whether they would then adopt a mammary stem/progenitor cell fate. This was first demonstrated with testicular cells isolated from a transgenic mouse model that allowed them to mark a particular mammary progenitor population (termed parity identified mammary epithelial cells-PI-MECs) [34, 35]. Remarkably, the testicular cells contributed to the outgrowths, and adopted all of the properties ascribed to normal PI-MECs including the persistence through multiple transplant generations, demonstrating they had not undergone terminal differentiation. These results were repeated with neuronal stem cells [24], lineage negative bone marrow cells [31], embryonic stem cells [32], and even human and mouse cancer cells [23, 28, 36].

These remarkable results were interpreted to mean that upon transplantation, the non-mammary cells were incorporated into mammary stem/progenitor niches during regeneration. Once inside the niche, they could function as fully competent mammary stem/progenitor cells. In addition, these results suggest that the properties we ascribe to stem cells should not be viewed as cell intrinsic features. Rather, “stemness” should be viewed as a cellular function, which is mediated by the niche/microenvironment in which the cell resides.

The ability of the microenvironment to control the cell fate of cancer cells is particularly intriguing as it demonstrates that a functional microenvironment/niche can rescue cellular function in genetically abnormal cells. This concept was also demonstrated using PI-MECs isolated from transgenic mice (WAP-INT3) that had aberrant notch signaling [30]. Within the transgenic hosts, the PI-MECs could not function as lobular progenitors. However, upon transplantation with wild-type mammary epithelial cells, their function was restored and they could produce lobules during pregnancy. From a regenerative medicine standpoint, this means that it is possible to repair dysfunctional tissues by repairing the microenvironment/niche. This could have important implications for regenerative medicine applications in neurological disorders where replacing neurons may not be reasonable, but repairing the microenvironment might be possible. The reverse is also true, as stem cells isolated from wild-type testicular cells could rescue alveolar development when combined with progesterone receptor null mammary epithelial cells [27]. Again, from a regenerative medicine perspective, this suggests it is feasible to rescue function of genetically abnormal tissues with genetically normal stem/progenitor cells.

It is still unclear what components of the mammary microenvironment are required for the cellular redirection described above. However, in a recent collaboration, we demonstrated that mammary ECM was sufficient to direct the differentiation of testicular and embryonic stem cells to form functional mammary glands in vivo [33]. These experiments were analogous to the ones highlighted above but instead of combining testicular and ESCs with normal mammary epithelial cells, the cells were simply mixed with soluble murine mammary ECM preparations isolated from fully developed adults. The result was a complete, functional mammary gland comprised entirely of the progeny of testicular or ESCs. Importantly, the mammary ECM also prevented teratoma formation by the ESCs, which formed large tumors when injected with vehicle alone in all cases. Again, these results have major potential implications for regenerative medicine, and provide support for the concept of using tissue specific ECM to provide scaffolding in regenerative medicine applications (discussed in Part II).

## Part II: Microenvironmental manipulation of cell fates for regenerative medicine

*“Early tissue and organ formation can be analogized to the formation of a hornet’s nest, which is a well-known example of a complex morphogenetic system. There is no genomic information or cell regulatory code that contains the “blueprints” for the construction of a “new” hornet’s nest. The nest architecture arises from the actions of thousands of hornets following simple instinctive rules. No biologist, and no hornet, can predict the location and exact shape of a given nest. Most importantly — the nest building process cannot be understood by the study of individual hornets or their sub-unit parts (eyes, legs, cells, proteins, genes).” Charlie D. Little*

### A brief history of hydrogels

Extracted ECM has established itself in the last few decades as a mainstay for the biomimetic culturing of cells. Original work in the field resulted in the establishment of polymerization and crosslinking methods for various naturally occurring materials including: collagen, fibrin, hyaluronic acid, chitosan, alginate etc. [37–42]. These biopolymers are capable of forming interactions with both the water they are dissolved in, and their neighboring molecules to generate a hydrate lattice structure termed a “hydrogel”. One ECM, collagen I extracted from rat-tails, is commonly used to coat plates for the attachment of many cell types. Since this technique was first reported in the 1950’s [40, 41], evidence has emerged demonstrating cells have more biologically relevant activities when grown in these contexts. Importantly, these initial experiments hinted that certain cell types required ECM molecules to maintain themselves in active 2D culture. These deductions subsequently revealed that indeed the culture of cell types such as embryonal carcinoma cells isolated from teratomas posed great difficulty in standard culture [43, 44]. Building from this, new supportive techniques were developed in order to culture and maintain these cell’s pluripotency, most notably the use of a fibroblast feeder layer originally described by Gail Martin in 1975 [45]. Later, these techniques were used for the successful isolation and culture of embryonic stem cells from both humans and mice [46–48]. The fundamental contributions of the fibroblast feeder layer were later determined to be several fold. Primarily, the fibroblasts operate by mechanically secreting ECM scaffolding enabling the attachment, survival, and vitality of these cells to a 2D culture vessel [49, 50]. Furthermore, the fibroblasts secrete key growth factors that signal cells to maintain their pluripotent state. While the definition of an ESC niche is still highly debatable, this culture technique ostensibly creates one, generating a microenvironment capable of maintaining a pluripotent state [51].

In an attempt to define the in-vitro embryonic niche, subsequent studies attempted to replace the feeder layers with ECM culture vessel coatings and media supplementation. Initially, Matrigel, an ECM extracted from the Engelbreth-Holm-Swarm (EHS) tumor grown in mice, was used to mimic the basement membrane-like composition of the embryonic environment [52–55]. This allowed for a feeder-layer free method of culturing pluripotent cells, with the caveat of batch to batch variability and issues with both viral and mouse protein contamination. In effort to define and simplify pluripotent cell culture many new techniques have emerged. These range from dynamic biopolymers and decellularirzed human fibroblast cultures to a single isoform of laminin or a truncated version of vitronectin [56–60]. This was further reinforced with the supplementation of a minimal media coupled with a set of growth promoters [57, 61]. These simplified systems of culturing a pluripotent cell is evidence of the basic components necessary to maintain an embryonic-like niche in-vitro. Thus, indicating that even with complex cell types such as iPSC and ESC, niche complexity is clearly dynamic with necessary signaling sometimes coming from only single sources. Moreover, without these systems in place, and without proper culturing technique, cells will continuously differentiate and undergo genomic instability [62, 63]. These data collectively highlight the vital nature of properly defining the microenvironment surrounding cells.

### Another dimension

While 2D studies have laid much of the ground work for understanding the biological activity of ECM on cells, the study of cells in their native 3-dimensions is necessary in order to fully understand their impacts. Evidence presented in the 1970’s demonstrated that cells cultured in 3-dimensions would make structures or spheroids that more closely resembled cells found in vivo [64, 65]. This technique, however, did not come to prominence until Mina Bissell’s laboratories experiments in the 1980’s. Here they demonstrated that 3D cultured mammary cells were capable of forming complex luminal structures similar to those found in vivo [66]. Since then, it has been demonstrated that growing cells in a 3D structure significantly alters the findings of similar 2D studies [67–69]. This seems to be especially true when discussing cancer cell growth and sensitivity to chemotherapeutics. When tumorigenic cells are placed into simple 3D ECM constructs, resistance to chemotherapeutics increases substantially [70–72]. While this subject is too broad for this review and has been covered elsewhere [73, 74], these data clearly indicate that the simple interactions with a 3D environment is sufficient to result in significant variations to cellular behavior. Thus, in this context, one could conclude that structured complex 3D ECM microenvironments would exhibit even further differences as compared to 2D culturing, possibly eliciting truly biomimetic behaviors.

In the quest to develop 3D tissue analogs, the current state of tissue engineering is dominated by synthetic alternatives. These approaches have been focused primarily on creating patentable methods to generate consistent, dissolvable, or stable structures. Often times the justification of a synthetic platform is due to the inherent variability found in natural materials [75, 76]. While this is certainly a complication, as mentioned earlier, work on natural materials has consistently been shown to generate more biomimetic responses. Furthermore, by its nature, the complex components that tissues are made up of are a requirement for proper function. Thus, simple synthetic systems are unlikely to elicit proper biomimetic responses. Evidence vindicating this perspective was first demonstrated by the introduction of Matrigel in the 1970’s. When used in vitro, it allows for the 3D growth of epithelial and endothelial luminal structures, while also enabling the study of the metastatic potential of cancer cells. Furthermore, due to its room temperature gelation characteristics, it is used extensively in vivo as a cellular “plug”, keeping cells where they are originally placed and also assisting in enhancing tumor take rates [77, 78]. A critical element of Matrigel is its complex and tissue like composition, which contains a diverse set of structural, functional, and signaling molecules. These molecules react in concert to define the space they occupy [54]. In contrast to engineering studies seeking to homogenize constructs, Matrigel offers the ability to mimic in 3D, the structural and biological function of a complex tissue. This complexity forms a 3D signature for each tissue, which is completed when cellular constituents are also included. It is important to note that tissues have a unique microenvironmental signature organ-to-organ and species-to-species that synergistically defines its function [79, 80]. Also, similar to the research examining the reaction of cells to mammary ECMs highlighted early in this review, cells placed into these complex 3D environments react in manners associated with the ECMs origin tissue [24, 25, 27, 31, 32]. Thus, a logical extension of these studies is the development of a 3D biomimetic system via the use of isolated ECM derived from model-specific source tissues.

### Tissue specific ECM

Controlling cell fate for tissue engineering applications and for the study of normal cellular behavior is of upmost importance. Accordingly, many studies have turned to tissue derived ECMs in an effort to faithfully recreate the target tissue in vitro. These systems have clear advantages, as they will contain the signaling cues necessary to properly guide cells, while also offering the opportunity to recreate the structural elements of the tissue. Several tissue engineering techniques have emerged to accomplish these goals that broadly fit into three categories: decellularized whole tissues, deconstructed/digested tissue ECM extracts, and constructs made of individual components found in the target tissue.

Decellularized tissues offer a unique opportunity to use intact scaffolding with all of the antigen presenting cellular components removed. Thus, one could repopulate a complete ECM with patient derived cells creating an immunologically compatible replacement to treat damaged or diseased tissues. Decellularization techniques predominantly use a detergent (e.g. SDS, NP-40, Triton-X etc.) to lyse and separate cellular components from the ECM. This allows for the preservation of the structural and tethered signaling molecules within the tissues microenvironment. This conserved state leaves behind the necessary signatures to properly instruct cells when reintroduced. As organ replacements are in limited supply, and with successful transplants still requiring constant immunosuppression, major work in the field has focused on whole organ engineering of hearts, lungs, kidneys and livers [81–83]. Early experiments on cardiac tissues demonstrated that cells can be completely removed and replaced with neonatal cardiac cells [84]. Of particular note, the cells would localize to appropriate areas and began to spontaneous contract in synchronization, indicating the remaining ECM was directing the cells placement and function. However, when whole hearts were seeded and tested, it resulted in an estimated ejection fraction of only 2% as compared to an adult rat [84]. Subsequent studies on decellularized human hearts carry technical limitations, due to the substantial increase in size of the organ from rats. However, it was reported that human cardiac tissues retain similar architectural structure once decellularized. Furthermore, it was also demonstrated that human mesenchymal stem cells, but oddly not human cardiac progenitors (hCPC) or human umbilical cord epithelial cells (HUVEC), would grow and repopulate sections of tissues removed from the organ [85]. Unfortunately, due to the size of human organs, proper decellularizing takes a significantly longer time, with less reliable outcomes. Furthermore, many of the remnant proteins still could carry some potential to illicit an immune reaction once transplanted [86–88]. Whether this is an issue for whole human organ decellularization still remains to be tested. Most importantly, initial transplantations of recellularized organs have demonstrated limited function leading to ultimate failure [89–91]. Nevertheless, it is promising to see that less complex decellularized human tissues such as skin, have been used for decades without any obvious immune rejection issues [92, 93]. While whole organ engineering could lead to the ultimate cure for diseases such as heart and lung failure, the complex nature of tissue organization presents many challenges before this techniques is ready for therapeutic use.

When considering potential alternative ECM based regenerative therapies, it is important to recognize that most diseased tissue have both a degradation of the structural elements of the ECM as well as the functional cellular components. Ultimately these losses result in the misdirection of cells within the destroyed ECM and the formation of scar tissue. This is particularly true of ischemic tissues, such as those found following a cardiac infarct, which tend to result in low or no-healing scars that participate in further organ dysfunction following the initial insult [94, 95]. Thus, regenerative therapies must consider how to properly initiate healing by signaling reparative cells to properly remodel the damaged tissues back to their original state. In an effort to accomplish this, Dr. Christman’s laboratory has produced ECMs derived from cardiac tissues [96–98]. The expectation being that these tissue-specific isolates from healthy ECMs will help to properly initiate the cascade of cellular infiltration and regeneration. Here they demonstrated that their isolations yielded ECM that mimicked the myocardium with a complex mixture of peptides as well as specific detection of GAG proteins. Furthermore, these tissue ECMs are capable of being tuned to suit the various handling demands necessary for operating room procedures with a 37 °C gelation temperature, tunable degradation rates, and the ability to be injected through a 27G catheter [96]. Importantly, when injected into a rodent heart, the gel allowed for the infiltration of both endothelial and smooth muscles cells [97]. The isolated ECMs also emulated the native environment by stimulating hCPCs to up regulate cardiac markers GATA-4 and MLC2V and VEGFR2 within only 4 days of culture [98]. However, there were significant composition differences when they performed these isolations on several different human hearts [99]. This indicates the importance of elucidating the specific variations, and describing the effective ECM combinations necessary to elicit reparative responses from cells. Furthermore, the fundamental approach of this style of engineering is to attempt to recreate specific tissues using extracted target-tissue ECM. However, a major limitation of using digested tissue ECMs is the random nature by which the matrices are reformed in the resulting engineered constructs. Thus, digested tissue ECMs when reconstituted lose much of their original mechanical properties. This often would necessitate modifications or additions to create structurally stable therapies. Additionally, due to the synergistic nature of the tissues microenvironmetal cues for proper cell direction, the exact signaling may not exist once the tissue derived ECM has gelled.

In order to fully understand the nature of these synergistic ECM interactions, researchers have performed high throughput analysis of mixtures of individual ECM molecules on stem cell fates [100, 101]. In these studies, various ECM molecules (e.g. collagen IV, fibronectin, nidogen, etc.) were mixed with various signaling molecules (e.g. FGF4, BMP4, LIF) and cell-to-cell interactive components (e.g. E-cadherin, jagged, EpCAM). Researchers then varied the mechanical properties of the hydrogels and the number of mouse embryonic stem cells per site to make 1024 unique conditions and studied their growth and differentiation [101]. These studies revealed that stiffness and lack of LIF would differentiate ESCs. Similarly the presence of BMP or FGF seemed to direct differentiation away from a pluripotent state. While these reductionist approaches could yield useful information about potential synergistic relationships amongst the several contributing factors in ECM, the simplified context could still miss the even bigger picture of complete 3D tissue formation. For example, it has been shown that changes in mammary gland ECM collagen architecture are responsible for pregnancy induced cancer prevention [102]. Further, these complex datasets are troublesome due to the extremely sensitive nature of pluripotent cells; simply changing the pressure on them can cause differentiation [103].

Similar to cell types where directed differentiation can be targeted through micro-environmental changes (MSCs [104], epithelial cells [105], myotubes [106]), neural stem cells are particularly sensitive to the substrate and matrix mechanical properties of their environment. Due to the unique nature of functional neurons maintaining G_0_ phase, it is critical to understand these environments to enhance survival.

It is now well understood that the brain microenvironment is primarily composed of proteoglycans, with the expression of basal membrane components: type IV collagen, laminins and fibronectin [107]. In general, these components are localized within three principle compartments/orientations: basal membrane lining cerebral vasculature, condensed perineuronal nets surrounding cell bodies, and neural interstitial matrix loosely arranged filling the parenchyma. While generally composed of identical ECM components, varying ratios or sub-components and tertiary structures determine their involvement in maintaining nervous system function.

Common in neurodegeneration disorders including Alzheimer’s, Parkinson’s, Huntington’s, amyotrophic lateral sclerosis, and multiple sclerosis, are the progressive loss of neurons and deterioration of nervous system structures. With the increasing of lifespan in the general population, these diseases are becoming more prevalent. While each disease has its unique etiology, they generally share some degree of protein aggregation, with evidence of this occurring within the extracellular matrix [108–114]. A number of studies have identified possible mechanisms of ECM degradation in neurodegenerative disorders, including matrix metalloproteinase activation [115], decreases in tissue inhibitors of metalloproteinase expression [116], aberrant expression of tissue plasminogen activators [117], and insult-induced neuro-inflammation [118].

Our comprehensive understanding of neurodegenerative disease-restructuring of brain microenvironment is lacking and the use of nervous system-derived ECM has yet to be extensively investigated, however, the potential therapeutic properties of ECM-based products is coming to light. Importantly, properly prepared engrafted ECM does not elicit an adverse immune response [119]. Millions of patients have been treated with ECM-based, FDA approved products in various tissues [120–123]. This evidence highlights the potential importance for recreation of biologically identical in vitro modeling for research, as well as for potential therapeutic purposes.

## Conclusion

The microenvironment is a complex 3D mixture of signaling molecules, interacting cells, and structural components. With each of these components playing a critical roll in healthy tissue, it is vital that we understand how their interplay works to identify methods to properly repair it when it is damaged in disease states. Furthermore, by thoroughly understanding the microenvironments participation in activating cell fate determination, we could better harness this tool for tissue engineering. Furthermore, with this knowledge we could also offer better detection methods to identify permissive environments that lead to diseases such as neurodegeneration, cancer, and cardiac disease.

## Abbreviations

ECM: Extra-Cellular Matrix
ESC: Embryonic Stem Cell
GAG: Glycosamino Glycan
hCPC: human Cardiac Progenitor Cell
HUVEC: Human Umbilical Vein Endothelial Cell
iPSC: induced Pluripotent Stem Cell
PI-MEC: Primary Mammary Epithelial Cell
